# Effect of Different Porous Size of Porous Inorganic Fillers on the Encapsulation of Rosemary Essential Oil for PLA-Based Active Packaging

**DOI:** 10.3390/polym16182632

**Published:** 2024-09-18

**Authors:** Raúl Cerdá-Gandia, Ángel Agüero, Marina Patricia Arrieta, Octavio Fenollar

**Affiliations:** 1 Instituto Universitario de Investigación de Tecnología de Materiales (IUITM), Universitat Politècnica de València (UPV), Plaza Ferrándiz y Carbonell 1, 03801 Alcoy, Spain; raucerg1@epsa.upv.es (R.C.-G.); anagrod@upv.es (Á.A.); 2 FAPERIN S.L. Av. de los Trabajadores, 27, 03430 Onil, Spain; 3 Departamento de Ingeniería Química Industrial y del Medio Ambiente, Escuela Técnica Superior de Ingenieros Industriales, Universidad Politécnica de Madrid (ETSII-UPM), C/José Gutiérrez Abascal 2, 28006 Madrid, Spain; 4 Grupo de Investigación: Polímeros, Caracterización y Aplicaciones (POLCA), 28006 Madrid, Spain

**Keywords:** polylactic acid, *Rosmarinus officinalis*, Diatomaceous earth (DE), Halloysite nanotubes (HNTs), active packaging, compostable

## Abstract

Essential oils are interesting active additives for packaging manufacturing as they can provide the final material with active functionalities. However, they are frequently volatile compounds and can be degraded during plastic processing. In this work *Rosmarinus officinalis* (RO) essential oil was encapsulated into Diatomaceous earth (DE) microparticles and into Halloysite nanotubes (HNTs) and further used to produce eco-friendly active packaging based on polylactic acid (PLA). PLA-based composites and nanocoposites films based on PLA reinforced with DE + RO and HNTs + RO, respectively, were developed by melt extrusion followed by cast-film, simulating the industrial processing conditions. As these materials are intended as active food packaging films, the obtained materials were fully characterized in terms of their mechanical, thermal and structural properties, while migration of antioxidant RO was also assessed as well as the compostability at laboratory scale level. Both DE and HNTs were able to protect the *Rosmarinus officinalis* (RO) from thermal degradation during processing, allowing to obtain films with antioxidant properties as demonstrated by the antioxidant assays after the materials were exposed for 10 days to a fatty food simulant. The results showed that incorporating *Rosmarinus officinalis* encapsulated in either DE or HNTs and the good dispersion of such particles into the PLA matrix strengthened its mechanical performance and sped up the disintegration under composting conditions of PLA, while allowing to obtain films with antioxidant properties of interest as antioxidant active food packaging materials.

## 1. Introduction

In the last decades, the continuing growth of an environmental concerns shown by society and its demands has propitiated that terms like circular economy, waste management, and sustainable consumption are the main research routes in the engineering and science fields. As a result of this, in the plastic sector, many works have focused on the study of bio-based and biodegradable polymers as alternatives to traditional plastics, particularly in short-term applications, to reduce the consumption of fossil-based resources and the plastics waste accumulation in the environment, avoiding micro-plastic generation. To produce fully sustainable biopolymeric formulations, natural additives are also required, such as vegetable oils as sustainable plasticizers or natural nanoclays as sustainable reinforcements, to produce high environmental performance composites or nanocomposites able to replace traditional plastics [1,2,3]. These naturally occurring materials also use to show some common characteristics, such as biodegradability, nontoxicity, biocompatibility and, in terms of processability, have been proven as a viable solution to substitute those obtained from nonrenewable sources in many industries [4]. As the food industry is one of the most involved sectors in the production of short-term plastics products, needing thousands of tons of plastics (traditionally obtained from fossil sources) to produce food packaging [5,6], the food packaging industry is one of the most involved sector focus on substituting the non-renewable and non-degradable polymers for sustainable biopolymeric based formulations [7].

Among all biopolymers, poly (lactic acid) (PLA) is the biobased and biodegradable polymer that has been positioned in the packaging industry as the most promising biopolymer to replace fossil-based polymers currently used in this field due to its ease of processing, availability in the market and economic competitiveness [6,8]. However, PLA possesses some disadvantages for food packaging, and this is why PLA-based composite and/or nanocomposites have gained industrial attention for food packaging purposes, since they are able to improve the mechanical and thermal properties of neat PLA. This industry has also been affected in the last years by a steadily increasing consumers demand for the use of more natural additives in processed food products [9].

In this sense, not only does the introduction of this eco-friendly material play a key role in the current global paradigm of the transition to a circular economy model, but also the possibility of providing these materials with active functionalities that allow the reduction of additives into the foodstuff. In this context, since lipid oxidation is one of the major reasons that compromise the food’s sensory performance which could also be harmful to consumers’ health, a new tendency in food packaging materials is to incorporate antioxidants into the packaging formulations instead of directly into the foodstuff [10,11]. In fact, active packaging is a technology thought to extend the food’s shelf life by promoting positive interaction between the packaging material and the foodstuff [12].

Therefore, besides the desirable characteristics for foodstuff packaging that biopolymers can supply, such as good processability, enough transparency, and adequate mechanical resistance; some natural essential oils (EOs) in eco-efficient composites are interesting due to their known active antimicrobial and/or antioxidant properties [13]. A feasible route to design active packaging materials is by the incorporation of active agents embedded into the polymeric matrix that would be part of the packaging system [14]. Thus, in concordance with the consumers’ increasing interest in non-processed and high nutritional food products, this has led to concepts such as active packaging totally linked with the sustainable concept in packed food [15]. In this context, essential oils (EOs) are naturally produced by plants and consist of complex lipids that are rich in biologically active compounds such as polyphenolic acids [16]. Some EOs obtained from thyme, oregano, sage, coriander, garlic, clove or rosemary have been well studied because of their effective antimicrobial and antioxidant properties [12,17,18]. In addition, most EOs are classified as Generally Recognized as Safe (GRAS) by the U.S. Food and Drug Administration [19].

As PLA is a biopolymer already integrated into the packaging industry, PLA, its blends, or biocomposites and bionanocomposites have been also successfully combined with different EOs. In this sense, Sharma et al. [20] concluded that the addition of Thyme and Clove essential oils on PLA/poly (butylen alipate-co-terephtalate) (PBAT) blend films processed by solvent casting has a great influence on the morphological and mechanical performance, while providing the material with antibacterial properties. Similar results were obtained in other work when Cinnamon and Eucalyptus essential oils were incorporated instead [21]. Meanwhile, Wang et al. [22] have blended PLA with Perilla essential oil by electrospinning, which resulted in a potential fresh-keeping packaging extending the shelf-life of chilled chicken by 12 days. Cheran et al. prepared nano film-coated paper based on PLA-nanocellulose incorporated with Thyme oil, Cinnamon oil, clove oil, and Rosemary oil and observed an increase in the hydrophobicity, while the materials showed active properties such as antimicrobial and insecticidal performance [23].

However, for the industrial production of PLA-based materials, they should be processed by melt-blending approaches, but as EOs are highly volatile, a high amount of them could be lost during the PLA-EO melt-processing, as it was already observed in PLA-EO formulations [24].

Among other essential oils, *Rosmarinus officinalis* L., an aromatic plant rich in phenolic compounds from the *Lamiaceae* family that originates from the Mediterranean region, is an interesting spice commercially available for use as an antioxidant. Rosemary extracts have been widely used in food preservation since they have shown their effectiveness in preventing oxidation and microbial contamination [25]. In this regard, there are some components present in rosemary extracts, i.e., carnosol, rosmanol, rosmarinic acid, and carnosic acid, that are effective for the antioxidative stabilization process for biopolymers [25,26].

Therefore, the introduction of EOs in the production process, due to their high volatility, usually involves a loss of some part of it, while the hydrophobic nature of the EOs could be adverse for a good dispersion in the PLA biopolymeric matrix, and consequently, the release of EOs is hardly homogenous and controlled. For this reason, encapsulation technology is a recurrent solution to protect active compounds from thermal degradation during PLA-based formulation processability and promote a dispersed phase in the polymeric matrix [12,27]. Regarding biopolymers reinforcement, nanoclays are the most prospective fillers due to their surface chemistry and morphology [28], which can increase PLA-based nanocomposite performance [12,18].

Halloysite nanotubes (NHTs) are naturally formed aluminosilicate (Al_2_Si_2_O_5_(OH)_4_·nH_2_O) clay with a characterized nanotubular hollow structure. On the external surface of the HNTs are located Si–O–Si groups, whereas the inner surface consists of an Al–OH array [29,30]. Due to this tubular shape with a negatively charged outside surface and a higher concentration of –OH groups in the inner lumen, HNTs can be easily dispersed into polymeric matrices. Commercially it can be found with a length of 50–500 nm, an external diameter of 20–200 nm, and an internal diameter of 10–70 nm [31]. HNTs have received considerable attention as nanofiller for enhancing the mechanical, thermal, and crystallization properties of several polymers such as PLA [32], chitosan [33], poly (ε-caprolactone) (PCL) [34], or polyamides (PA) [30,35]. As HNTs belong to GRAS kaolin clays for food contact materials owing to their non-toxicity and biocompatibility, they have been also employed as an encapsulation system for active packaging applications [36,37,38].

Diatomaceous earth (DE) is also a naturally occurring clay that can be found in geological deposits. DE is basically fossilized skeletons of unicellular microorganisms living in either fresh or seawater all over the world. Although there exist around 30,000 diatom species, all of them present a silica shell with different structures and regularly organized slits or pores [39,40]. These microstructures are composed mainly of amorphous silica (SiO_2_·nH_2_O), have a large effective surface area, above 200 m^2^ g^−1^, and a high absorption capacity due to their nanoporous with a range size from 10 to 1000 nm [41,42]. DE is a well-recognized encapsulating material with applications in filter, gas sensors, or drug delivery systems [30,43]. They have also been used as reinforcement/encapsulating particles in green composites based on PLA [44,45].

The purpose of this study is to reinforce PLA matrix with two natural clays, with different size ranges, as encapsulating systems for food active film packaging production by a melt-extrusion approach. For this, HNTs (nanoparticles) and DE (microparticles) were loaded with an active agent and then incorporated into the PLA matrix to produce active films. *Rosmarinus officinalis* essential oil (RO) was the active agent selected due to its local abundance and for its widely reported active (antioxidant and antibacterial) properties [46,47]. The HNTs nanoparticles were characterized by DLS, while both HNT nanoparticles and DE microparticles were observed by transmission electron microscopy (TEM). PLA is an elsewhere known biopolymer, and the contribution of the loaded HNTs and DE particles was studied in terms of structural, mechanical, and thermal properties by means of scanning electron microscopy, tensile test measurements, differential scanning calorimetry, and thermogravimetric analysis. The antioxidant activity of the RO and the effectiveness of the two encapsulating systems were analyzed by measuring the antioxidant activity of the films exposed to a fatty food simulant through the measurement of DPPH scavenging activity, while the disintegrability under composting conditions was conducted at a laboratory scale level to corroborate the biodegradable character of the developed materials.

## 2. Materials and Methods

### 2.1. Materials

The PLA used was Ingeo™ Biopolymer 2003D commercialized in pellets form by NatureWorks LLC (Minnetonka, MN, USA). This PLA grade has a melt flow rate (MFR) at 210 °C of 6 g 10 min^−1^ and a true density of 1.24 g cm^−3^, which makes it adequate for suitable for melt blending process. *Rosmarinus officinalis* essential oil (RO), CAS 8000-25-7, was purchased from Esencias Martinez Lozano S.A. (Murica, Spain). According to the producer, this EO has a density of 0.892–0.910 g cm^−3^, comes from plant leaves, and its principal chemical compounds are α-pinene + α-thuyene (18–26%), 1-8-cineole (17–25%), camphre (12.5–22%), and camphene (8–13%). Halloysite nanotubes (HNTs) in powder form, CAS 685445, were provided by Sigma-Aldrich S.A. (Madrid, Spain). According to specifications, HNTs density is 2.53 g cm^−3^, while nanotubes diameters are between 30 and 70 nm, with a length of 1–3 µm, an effective surface area of 64 m^2^ g^−1^ and a pore volume of 1.26–1.34 mL g^−1^. Meanwhile, Diatomaceous earth (DE), CAS 68855-54-9, with a density of 2.36 g cm^−3^, is also distributed in powder form by Sigma-Aldrich S.A. (Madrid, Spain). This product is a mixture of diatoms species shells with a wide variety of particle sizes and geometric shapes, but disc and tubular forms are predominant, with an average particle size of around 125 µm. The ethanol with 99.8% purity, and The radical 2,2-diphenyl-1-picrylhydrazyl (DPPH) were also obtained from Sigma-Aldrich S.A.

### 2.2. Essential Oil Encapsulation

**Figure 1:**
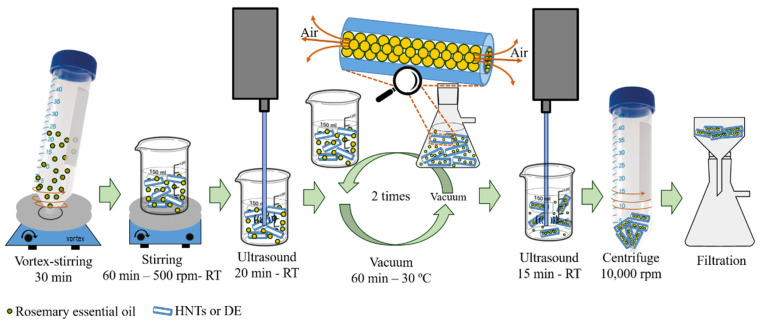
Schematic representation of the HNTs or DEs encapsulation process with RO.

Firstly, to eliminate residual moisture due to the hydrophilic nature of these silica particles, HNTs and DE were dried at 150 °C for 3 h. Then the RO was encapsulated into HNTs or DE particles using a vacuum procedure according to previous reports [28,37] with slight modifications. Briefly, a solution of rosemary essential oil (RO) at 25 wt.% in ethanol was prepared and fully mixed by vortex-stirring for 30 min. Subsequently, each encapsulation material (HNTs or DE) was separately added to the RO solution at a weight ratio 1:0.2 and stirred at room temperature at 500 rpm for 60 min followed by ultrasonic homogenization in a Thermo Fisher Scientific Q125 (Waltham, MA, USA) at 10% of amplitude equipped with a tapered microtip CL-18 for 20 min in an ice bath, while an on/off pulse mode was chosen to prevent the overheating of the system. The resultant suspensions were subjected to vacuum for 1 h at 30 °C, and then cycled back to atmospheric pressure to replace the entrapped air in the particles lumens with the oil solution. Afterward, both mixtures were again homogenized by ultrasonication for 15 min. Finally, the remaining liquid was separated by centrifugation, washed with ethanol to remove the unencapsulated RO from the external HNTs and DE, and finally filtered to obtain a powder. In Figure 1 there is a schematic representation of the encapsulation process. Before processing with PLA matrix, the loaded HNTs and DE particles were dried in an air-circulating oven at 80 °C for 12 h [31].

### 2.3. Nanoparticles Sizes Characterization

The hydrodynamic particle sizes of the HNTs in water (1 mg mL^−1^) were measured at 20 °C with a dynamic light scattering (DLS) analyzer (BeNano 90, Nane Vita, Madrid, Spain) equipped with a 50 mW solid-state laser beam at 671 nm and with a detection angle of 90°. The reported particle size was the average value of at least five consecutive runs.

Transmission electron microscopy (TEM) images of HNTs and DE containing the RO essential oil were obtained by transmission electron microscopy (TEM, JEOL JEM-1010) operating at 100 kV. One droplet of DE or HNT aqueous suspension was deposited on carbon-coated copper grids and allowed to dry at room temperature for 25 min before observations.

### 2.4. Films Processing

Once the HNTs and DE particles were loaded with the RO, namely HNTs + RO and DE + RO respectively, they were used to reinforce PLA. Thus, PLA was loaded with 1, 3 and 5 wt.% of HNTs + RO and DE + RO. The as-received PLA pellets were previously dried at 60 °C for 12 h in a dehumidifier MDEO from Industrial Marsé S.L. (Barcelona, Spain). The selected HNTs + RO and DE + RO contents were chosen since some authors have reported that over a 3–5% nanoclays content in biopolymer composites, some filler aggregates can occur, and general material performance is compromised [48,49]. In addition to PLA-HNTs + RO and PLA-DO formulations, pristine PLA film and formulations with only RO incorporated at three concentrations (0.5, 1, and 3 wt.%) were also produced for comparison and labeled as PLA-RO 0.5, PLA-RO 1, and PLA-RO 3 respectively.

The different amounts of materials were mechanically mixed and were then melt-compounded in a twin-screw extruder from Construcciones Mecánicas Dupra S.L. (Alicante, Spain). The rotating speed was set at 20 rpm and the temperature profile from the feeding hopper to the extrusion die was: 185 °C, 190 °C, 195 °C, and 200 °C. After the extrusion, materials were pelletized in an air-knife unit for further processing in a cast-film extruder from Eutotech S.A. (San Martino in Riu, Italy) with a 200–205 °C temperature profile from the feeding to the head. The extrusion speed was 20 rpm and the calendar and drag speed were adjusted to obtain an average thickness of 30–50 µm approximately [50]. The resultant film rolls were attained and stored at room temperature for 15 days before testing.

### 2.5. Film Characterization

#### 2.5.1. Scanning Electron Microscopy

The surface morphology of the cross-section of cryo-fractured samples using liquid N_2_ was evaluated by JEOL JSM-6300 scanning electron microscopy (SEM) (JeolUSA Inc., Peabody, MA, USA). The samples were previously sputtered with a gold-palladium alloy in an EMITECH sputter coating SC7620 model from Quorum Technologies, Ltd. (Lewes, UK).

#### 2.5.2. Mechanical Test

Tensile properties of the films produced were determined in a universal test machine ELIB 30 from S.A.E. Ibertest (Madrid, Spain). The tests were carried out at room conditions following the guideline of ISO 527-2:2012 [51] for rectangular samples sizing 10 × 150 mm^2^, the selected load cell was 5 kN and a crosshead speed of 2 mm min^−1^. At least five samples of each formulation were tested.

#### 2.5.3. Thermal Properties

##### 2.5.3.1. Differential Scanning Calorimetry

To study the thermal transition of the different formulations, DSC analyses were carried out in a Differential Scanning Calorimeter DSC 821 from Mettler-Toledo Inc. (Schwerzenbach, Switzerland). Samples with an average weight of 6–8 mg of each formulation were placed in an aluminum-sealed 40 µL crucible and subjected to a single-step cycle from 25 °C to 300 °C and run at a range of 10 °C min^−1^ in an air atmosphere. Crystallinity degree (χ_c_%) was calculated following Equation (1):

(1) $$\chi_{C}=\left[\frac{\left|{∆H}_{m}|-{|∆H}_{CC}\right|}{{∆H}_{m}^{0}\left(1-w\right)}\right]\times 100$$

where ${∆H}_{m}$ (J g^−1^) is the melting peak enthalpy, ${∆H}_{CC}$ (J g^−1^) is the cold crystallization peak enthalpy, ${∆H}_{m}^{0}$ (J g^−1^) is the theoretical value of the melting enthalpy of a fully crystalline PLA polymer, reported in the literature as 93.7 J g^−1^ [52], and *w* represents the weight fraction of PLA in the sample.

##### 2.5.3.2. Thermogravimetric Analysis

The thermal stability of the formulations was determined by thermogravimetric analysis (TGA) using a thermobalance TGA PT1000 from Linseis Inc. (Selb, Germany). For each formulation, a sample with an average weight of 5–10 mg was placed in standard alumina 90 µL crucibles and subjected to a thermal program from 40 °C to 800 °C with a heating rate of 20 °C min^−1^.

#### 2.5.4. Antioxidant Activity

The antioxidant activity of the RO released from films into a food simulant in this work was determined by the DPPH inhibition method [10,14]. With this aim, samples of 15 mm × 15 mm of each formulation were submerged in 4.5 mL of fatty foodstuff simulant (Simulant D1, 50% *v*/*v* ethanol). The DPPH radical scavenging activity (RSA) of the simulant D1 was measured after 10 days at 40 °C in contact with the active films. For this purpose, 2.7 mL of the food simulant was mixed with 3 mL of 2 mM DPPH solution and maintained in darkness for 15 min at room temperature. The DPPH absorbance was recorded at the wavelength of 517 nm using a UV–VIS Varian Cary spectrophotometer (Varian, Palo Alto, CA, USA). The antioxidant capacity of the films was expressed as a percentage of inhibition of DPPH radical by the determination of the radical scavenging activity (RSA) using the Equation (2):

(2) $$RSA\;(\%)=\frac{A_{0}-A_{t}}{A_{0}}\cdot 100$$

where $A_{0}$ is the absorbance of the control and $A_{t}$ is the absorbance of the food simulant after the migration test and its reaction with DPPH. At least three different samples migrated from each film were measured.

#### 2.5.5. Disintegration under Composting Conditions

Following ISO 20200 [53], the disintegrability of a polymer based material can be considered as the mass loss of a sample buried in a controlled compost soil. To this end, square samples of 25 × 25 mm^2^ of each film formulation were placed in a bioreactor, prepared as indicated by the cited standard, and kept at 58 °C and water content in the soil of around 55%. The disintegration process under composting conditions was carried out during four weeks. Every week (7 days) samples were unburied, washed with distilled water, and dried at 40 °C for two days to be reweighed in an analytic thermobalance. The weight loss percent during the biodegradation process was calculated using Equation (3):

(3) $$Weight\;loss\;(\%)=\frac{W_{0}-W_{t}}{W_{0}}\cdot 100$$

where $W_{0}$ is the weight of the sample before disintegration and $W_{t}$ represents the weight after a disintegration time t.

#### 2.5.6. Statistical Analysis

The significant differences in mechanical, thermal and antioxidant properties were statistically assessed at 95% confidence level using a one-way analysis of variance (ANOVA) according to Tukey’s test employing OriginPro2018 software (OriginLab, Northampton, MA, USA).

## 3. Results

### 3.1. Particles Characterization

#### 3.1.1. Dynamic Light Scattering

**Figure 2:**
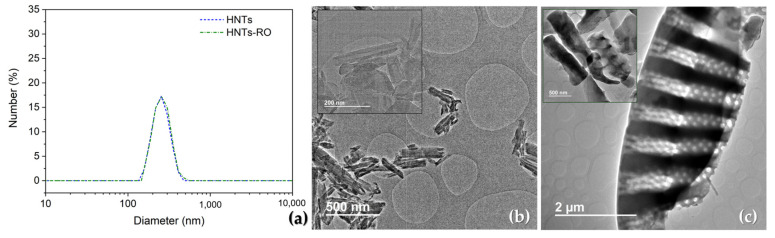
(**a**) DLS of HNTs and HNTs + RO as well as TEM images of (**b**) DE microparticles and (**c**) HNTs nanoparticles.

The hydrodynamic sizes of the pristine HNTs and HNTs encapsulating RO were determined by DLS, which showed a monomodal size distribution showing no significant differences between them with well-defined peaks between 155 nm to 410 nm (Figure 2a) centered at 254 nm in the case of HNTs and at 257 nm for RO loaded HNTs. However, the DLS technique is designed to calculate the hydrodynamic diameter of spherical nanoparticles, and thus, to corroborate the tubular shape as well as the actual dimensions of HNTs, TEM observations were also conducted. Additionally, as DE are microparticles, it was not possible to measure the particles through DLS and they were directly observed by TEM.

#### 3.1.2. Transmission Electron Microscopy

The TEM images of the obtained HNTs and DE after the encapsulation treatment are shown in Figure 2b,c. As expected, the presence of RO could not be distinguished as no significant differences were observed between either HNTs or DE before the encapsulation process (without EO, not shown) and the HNTs and DE containing the RO. Although HNTs (Figure 2b) tended to aggregate in the TEM grid as a consequence of their high hydrophilicity [54], the TEM analysis confirmed the dimension of the nanotubes observed by DLS and from the zoom image (Figure 2b) of HNTs the tubular structure can be clearly seen. In the case of DE (Figure 2c), it showed microparticles with a high characteristic porosity that showed different shapes and a wide variety of sizes in good accordance with previous work [41].

### 3.2. Films Characterization

#### 3.2.1. Visual Appearance

**Figure 3:**
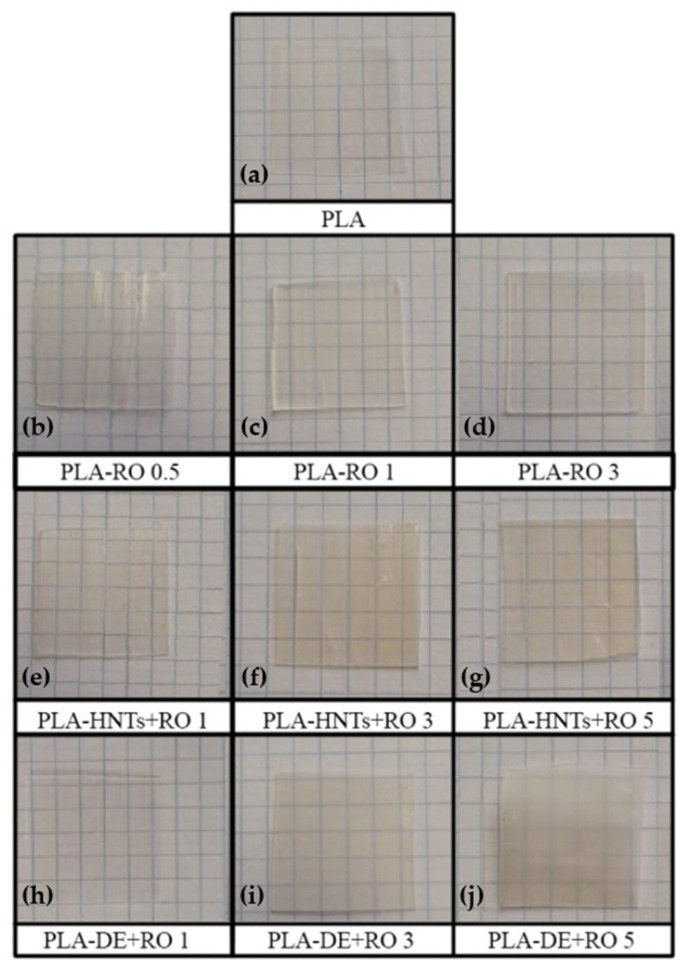
Visual appearance of the obtained films. (**a**) PLA; (**b**) PLA-RO 0.5; (**c**) PLA-RO 1; (**d**) PLA-RO 3; (**e**) PLA-HNTs + RO 1; (**f**) PLA-HNTs + RO 3; (**g**) PLA-HNTs + RO 5; (**h**) PLA-DE + RO 1; (**i**) PLA-DE + RO 3; and (**j**) PLA-DE + RO 5.

Figure 3 shows the visual appearance of the produced neat PLA film, Rosemary essential oil (RO) incorporated PLA films (PLA-RO), and PLA films loaded with Diatomaceous earth (DE) microparticles and Halloysite nanotubes (HNTs) encapsulating Rosemary essential oil (PLA-NHTs + RO and PLA-DE + RO films). The PLA film sample, at the top of the image (Figure 3a), is characterized by a high transparency. The three images below correspond to the film of PLA with the direct incorporation of RO (Figure 3b–d). The concentration of this essential oil added to the PLA matrix in this study seems low enough not to affect significantly the transparency of the material. Continuing, in the middle row of the figure are the images of the PLA films with RO encapsulated into HNTs at different contents (Figure 3e–g). The presence of the RO loaded HNTs nanoparticles is observable even at low concentrations, showing the PLA-HNTs + RO 1 certain yellowing compared with neat PLA film. This is more evident when the HNTs + RO is increased, acquiring besides a greater opacity at higher contents. Finally, when the RO is encapsulated in the DE the modification of the optical appearance of the PLA is more marked (Figure 3h–j) and related to the loss of transparency, getting more opaque aspect when more DE + RO amount is added.

#### 3.2.2. Scanning Electron Microscopy

**Figure 4:**
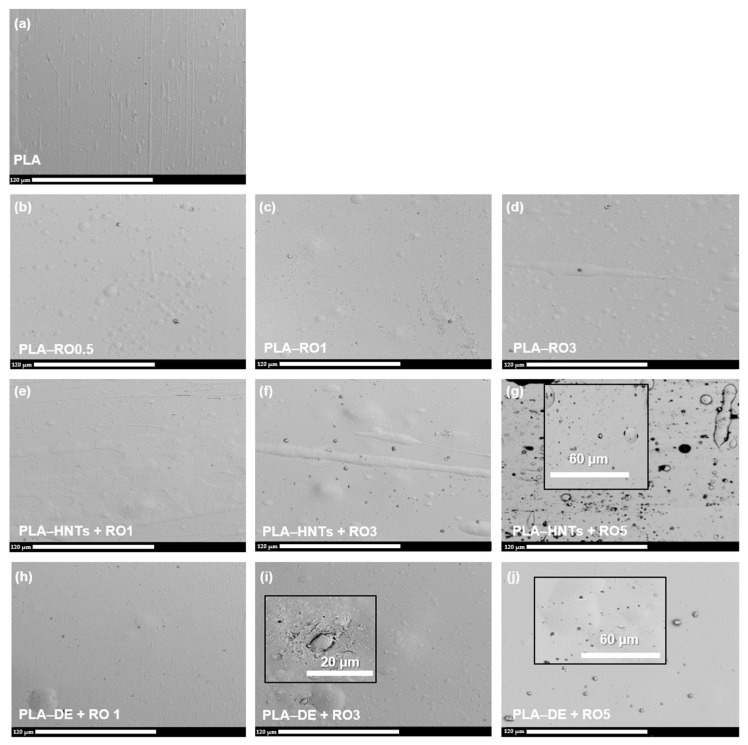
SEM images of the cryo-fracture section of the obtained films. (**a**) PLA; (**b**) PLA-RO 0.5; (**c**) PLA-RO 1; (**d**) PLA-RO 3; (**e**) PLA-HNTs + RO 1; (**f**) PLA-HNTs + RO 3; (**g**) PLA-HNTs + RO 5; (**h**) PLA-DE + RO 1; (**i**) PLA-DE + RO 3; and (**j**) PLA-DE + RO 5.

**Figure 5:**
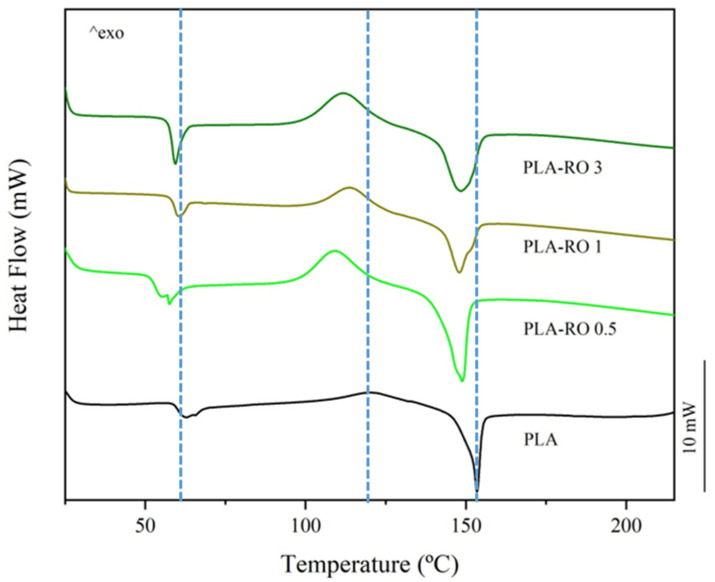
DSC first heating scan of the neat PLA and PLA with direct addition of RO films.

Figure 4 shows the SEM micrographs of the cross cryo-fractured sections of the films developed here. From the cryo-fracture surface of the neat PLA film (Figure 4a), it can be seen that the typical rigid brittle and smooth fracture surface of PLA [10,54]. The incorporation of essential oil in 0.5 wt.% practically did not produce changes on the fracture surface (Figure 3b). Meanwhile, higher contents of rosemary essential oil of 3 wt.% (Figure 4c) and 5 wt.% (Figure 4d) produced homogeneous cryo-fracture surfaces with more plastic deformation, producing a somewhat plasticization effect as was already observed in PLA blended with other essential oils, such as limonene [24,55], carvacrol [56], thymol [18], or cinnamaldehyde [18]. The no apparent phase separation suggests good compatibility between RO and PLA matrix.

In the case of nanocomposites and composites, it is widely known that the particle dispersion is one of the major challenges to obtaining improved properties in the final material, particularly when using highly hydrophilic nanoparticles such as HNTs, which tend to form aggregates in the polymeric matrix when processed by conventional melt blending approaches [54,57]. From the SEM observations, it can be seen that both particles appeared well dispersed in the melt-blended polymeric matrix, which influenced that the films were still transparent after the addition of the HNTs and DE particles (see Figure 3).

The fracture surfaces of HNTs-based nanocomposites (Figure 4e–g) and DE-based composites (Figure 4h–j), showed that both particles reduced the smoothness and the materials showed more roughness as the particle amount increased in the film formulations.

In HNTs-based nanocomposites, some small white spots on the cross-section were observed which became denser with increasing HNTs concentration in the formulation from 1 wt.% (Figure 4e) to 3 wt.% (Figure 4f), while micro-voids are present in the nanocomposite with the higher concentration used here of 5 wt.% (Figure 4g). Similar findings were obtained by Rojas-Lema et al. who observed that when PLA was loaded with HNTs above 5 wt.%, the HNTs pull-out due to their poor interfacial adhesion with the PLA polymeric matrix which is intensified as a consequence of high HNTs loadings which can form agglomerates [57]. Similarly, De Silva et al. also observed an increase in the pores and microvoids formation with increasing HNTs content, particularly at PLA loading levels higher than 5 wt.%, and attributed these findings to an increasing detachment of the HNTs from the PLA matrix due to HNTs aggregation which ultimately leads to an increasing phase separation [58]. DE-based nanocomposites also showed the increasing density of the small white spots on the cross-section with an increasing amount of DE in the formulation. While the PLA loaded with 1 wt.% (Figure 4h) shows a homogeneous and regular matrix, for composites with DE higher loadings of 3 wt.% it is possible to identify individual DE particles and the surroundings of these microparticles show a small gap between them and the PLA polymeric matrix (see zoom image in Figure 4f). Similar findings have been already observed in a previous work [41]. Conversely, micro-voids are present in the nanocomposite with the higher concentration used here of 5 wt.% (Figure 4g), these unexpected results, in which better compatibility between the PLA polymeric matrix and DE at higher loading levels (5 wt.%) could be related to the higher amount of RO in this formulation that improves the compatibility between the microparticles and the polymeric matrix.

#### 3.2.3. Mechanical Characterization

Tensile test results of the manufactured cast-extruded films are gathered in Table 1. Firstly, it can be observed that the neat PLA film showed the characteristic brittleness of this polymer, with a tensile elastic modulus (*E*) of 3700 MPa, tensile strength at yield (σ_y_) of 38.5 MPa and a very low elongation at break (ε_b_) of 2.3% [59]. It can be realized that the direct addition of a small percent of RO (0.5 and 1 wt.%) into the film formulation exerts similar and no significant (*p* > 0.05) variations in the strength at yield and elongation at break values, remaining around 38–39 MPa and 1.5%, respectively, through a small increase in the elastic modulus (*E*), reaching values of around 4000 MPa. But, when the RO content increases to 3 wt.% (PLA-RO 3), certain plasticization of the PLA matrix is produced with an increment in the flexibility of the films and a decrease in tensile strength. Specifically, σ_y_ values significantly (*p* < 0.05) decrease to 26 MPa, while elongation at break significantly (*p* < 0.05) enhances to 3.6%. As a consequence, the elastic modulus returns close to neat PLA values, reaching even significantly (*p* < 0.05) lower values. This reduction of the elastic modulus is due to the directly related decrease in strength and the increase in ductility, since the tensile modulus represents a coefficient of these parameters in the elastic behavior of the material [60]. The plasticization effect that EOs produce in PLA matrices has already been studied and reported in other works [18,24,61]. Some authors have concluded that the incorporation of EOs may interfere with polymer chains, reducing the intermolecular forces and, thus, acting as a plasticizer with a consequent improvement of the chain mobility and the flexibility of the material [12,24].

With regards to the PLA film with RO encapsulated into the two different particles (HNTs and DE), it was evidenced that each type of particle employed has a different effect. In this sense, the incorporation of 1 wt.% of HNTs induces a slight but significant (*p* < 0.05) increase in the elongation at break, and a significant (*p* < 0.05) reduction in the tensile strength value, reaching values close to that of PLA-RO 3, indicating that the RO essential oil role is more relevant than the HNTs particles, resulting in a plasticization of the polymeric matrix. However, HNTs seem effective in protecting the RO from thermal degradation since for a lower amount supplied, the results are very similar. The HNTs contribution is noticeable for the intermediate content formulation, PLA-HNTs + RO 3, which showed the most balanced mechanical performance with a tensile strength, and elastic modulus close to that of PLA while somewhat improving of the elongation at break. Generally, when a nanofiller is introduced and well dispersed in a polymeric matrix, due to its extensive surface area able to interact with the polymeric matrix, a greater difference in the material properties is caused [62]. Instead, at the highest HNTs content, which is in PLA-HNTs + RO 5, the enhanced parameters tended to decrease. The results obtained here are totally in concordance with the structural defects shown in SEM as well as with the literature. For instance, Rojas-Lema et al. [57] found similar results in plasticized PLA/OLA films with HNTs contents above 3 phr. Additionally, another study has reported that 3 wt.% of HNTs resulted in the optimal mechanical properties in PLA-based films, forming agglomerates if this content is exceeded [63].

On other hand, the reinforcement effect of the DE filler of the composite film is noticeable even with the lowest content. In terms of mechanical performance, PLA-DE + RO 1 resulted as the optimum formulation of composite with these microparticles added, with values of 47 MPa for tensile strength, 4.85% for elongation at break, and 4110 MPa for elastic modulus. This is indicative of a more homogenous dispersion of the DE in the PLA matrix compared with HNTs. Besides, the good compatibility between DE particles and PLA has been reported by other authors [44], highlighting that the high porosity of the diatoms allows polymer chains to enter and go out, providing a good particle/matrix adhesion. With a higher content of encapsulating filler (PLA-DE + RO 3) an interesting phenomenon seems to occur. At this amount DE tends to self-aggregate and form a dispersed phase [49,64]. Hence, the tensile properties of PLA-DE + RO 3 are significantly (*p* < 0.05) reduced with respect to PLA-DE + RO 1, with a strength value of 39.2 MPa and elongation at break of 1.6%, whereas rigidity still grow, leading to a modulus of around 4550 MPa. Finally, due to the low retention capacity of DEs when the addition of this filler with RO up to 5 wt.% (i.e., PLA-DE + RO 5), it seems that the already mentioned high porous microstructure of the DE particles is not able to retain as much RO essential oil [65], and this means a major amount of essential oil at the particles surrounding. This excess of essential oil is presumably released slowly from the DE particles and interacts with the PLA chains, plasticizing the material and leading to a better compatibility between DE and the polymeric matrix.

#### 3.2.4. Thermal Properties

##### 3.2.4.1. Differential Scanning Calorimetric

Differential scanning calorimetric (DSC) analyses were carried out to study the different thermal transitions of the PLA-RO films and the influence of the encapsulation of the RO in HNTs and DE, and Table 2 lists the main thermal parameters from DSC of the PLA-RO, PLA-HNTs + RO, and PLA-DE + RO developed films as well as the neat PLA film. The glass transition temperature T_g_, melting temperature T_m_, and crystallinity grade ($\chi_{c}$) of the PLA were at 60.3 °C, 153.5 °C, and 12.0%, respectively.

Concerning the direct addition of RO, essential oil molecules interact directly with the polymer chains and the thermal parameters modifications are directly related to the RO content. In Figure 5, the DSC first heating thermograms corresponding to neat PLA and PLA-RO at different percentages are plotted. Therefore, it is possible to observe a similar and no significant decrease in the T_g_ (*p* > 0.05) and a small but significant decrease in T_m_ (*p* < 0.05) for all the PLA-RO films but a higher reduction in crystallinity $(\chi_{c}$) for a greater amount of essential oil, down to a 6.7% for PLA-RO 3. A significant (*p* < 0.05) reduction in the cold crystallization temperature (T_cc_) was observed for all samples containing the RO either non-encapsulated or encapsulated in HNTs and DE. This behavior could highlight the compatibility between the RO and both particles, HNTs and RO, with the polymeric matrix, where as a result of the decrease in T_g_, a subsequent lowering in the transport barrier for crystallization takes place, leading to a lower cold crystallization temperature, as it was already observed for PLA-Limonene blends [24]. These changes in thermal properties are in concordance with the tensile test results previously discussed. It is well studied that essential oils provide a plasticizing effect on PLA matrices, decreasing the glass transition temperature and crystallinity due to the increase in the free volume and chain mobility [61].

**Figure 6:**
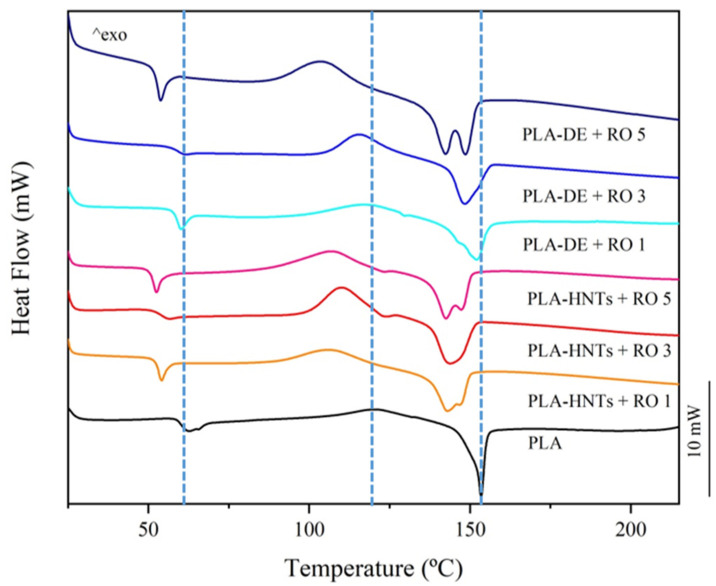
DSC first heating scan of the neat PLA, PLA-HNTs + RO, and PLA-DE + RO films.

With the encapsulation of the RO in HNTs particles the modification in the DSC transition temperatures is close to those obtained with the direct addition of the essential oil to PLA (Figure 6). The T_g_ and T_m_ values decreased from 60.3 °C to 58–59 °C and from 153.5 °C to 147–148 °C, respectively, whether a 1–3 wt.% of RO or 1–3 wt.% of HNTs + RO are added, indicating that the essential oil is mainly what modifies the polymer matrix. In fact, the poor interactions between HNTs and PLA have been reported by Montava-Jorda et al. [54], while here it seems that the RO is increasing the interaction between PLA and HNTs. For the PLA-HNTs + RO 5, the inherent increment of encapsulated vegetable oil follows the tendency and produces a higher reduction but not significant (*p* > 0.05) of the T_g_ value (56.6 °C). Attending to the degradation of crystallinity $(\chi_{c}$), a remarkable decrease was obtained, with values around 1%. This is a consequence of the increment of the crystallization enthalpy (${∆H}_{cc}$), raised to 26.7 J g^−1^, which is almost triple that of the neat PLA (9.5 J g^−1^). This could be attributed to the fact that HNTs avoid the crystallization of the PLA during the cooling of the material during the film-forming process and this is why it crystallizes during the DSC heating. Additionally, there is a reduction in the T_cc_, which can be attributed to the presence of less perfect crystals as reported by Chen et al. [66] due to the presence of HNTs. On the other side, Lee et al. studied the encapsulation of thyme oil in HNTs and concluded that the thyme oil molecules were successfully loaded in the lumen of the nanoclays without producing any modification to the nanotubes [9]. Less perfect crystals, which start to form at a lower temperature, and a considerable amount of oil trapped in the HNTs agglomerates, induce a longer time for full crystallization, which causes the high reduction in the crystallinity already mentioned. Besides, this is totally in concordance with the double melting peak that appeared in the PLA-HNTs + RO 5 thermograph (Figure 6), in which less perfect crystals melt at significantly lower (*p* < 0.05) temperatures, while the most perfect crystals melt at higher values.

Using DE + RO as an encapsulating filler material did not suppose a significant difference in terms of the transition temperatures compared with HNTs. One can see (Figure 6) how, for PLA-DE + RO 1 and PLA-DE + RO 3, the advance of the glass transition and melting temperatures is practically the same as that of the HNTs encapsulating particle counterpart, although somewhat less pronounced. As occurs with PLA-HNTs + RO 5, the high content of essential oil in PLA-DE + RO 5 translates into an even greater advancement of temperatures, with no significant (*p* > 0.05) change in the T_g_ of 58.0 °C and a significant reduction of the T_m_ (*p* < 0.05). It can be concluded that neither micro- nor nanoclays modify the polymer structure but rather it is the RO that does it. Nevertheless, the release mechanism of RO from the particles seems to play a significant role in the final crystallinity of the material. The $\chi_{c}$ of the PLA-DE + RO 3 is around 7%, which is 42% lower than that of neat PLA. However, for the PLA-DE + RO 5 films, this value is increased to 22.4%, almost twice that of neat PLA. This tendency is parallel to the results obtained in the tensile test, with a reduction of ductility and strength for a 3 wt.% DE + RO and then an enhancement of these parameters for the 5 wt.% DE + RO. It can be hypothesized that the small phase rich in essential oil formed in PLA-DE + RO 3 due to a complete dispersion within the polymeric matrix interrupts the continuity and inhibits the crystal growth. Whereas with 5 wt.% of DE + RO, the presence of a higher amount of essential oil interacting with PLA chains allows the formation of a second population of crystals, which is also evidenced by the double peak in the melting enthalpy curve.

##### 3.2.4.2. Thermogravimetric Analysis

**Figure 7:**
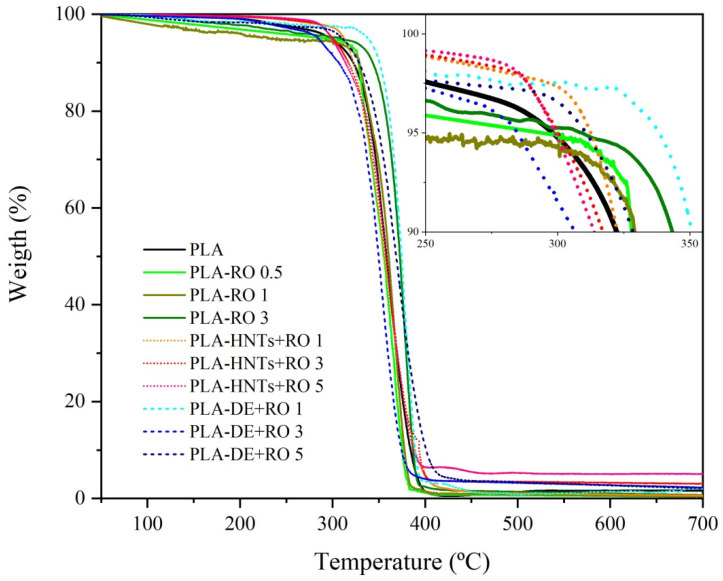
TGA curves and DTG (zoom image) of films.

TGA assays were conducted to study the influence of the RO and the use of HNT_S_ and DE as encapsulation fillers on the thermal stability of the PLA films. The TGA curves for PLA, PLA-RO, PLA-HNTs + RO, and PLA-DE + RO at different loading levels are shown in Figure 7, whereas the main values obtained from these curves are summarized in Table 3.

It can be observed that all the films presented a single-step pattern of mass loss. This similarity in the thermal stability of the materials developed here and the PLA was as expected for PLA [22]. The onset degradation temperature of PLA, meaning when 10% of mass loss occurs ($T_{10\%}$), was at ~322 °C and the maximum thermal degradation temperature ($T_{deg}$) was above 378 °C.

The direct addition of a small percent of RO provided a thermal stability improvement, and it is more noticeable for the highest contest, resulting in a $T_{10\%}$ at ~343 °C, significantly higher (*p* < 0.05) for PLA-RO 3 as a consequence of the thermal protection of an antioxidant compound on the polymeric matrix provided by the RO. In this context, similar delays in the onset degradation temperature of PLA have been observed in PLA added with garlic acid [50] and PLA added with catechin [14], attributing this enhancement to the intrinsic antioxidant activity of the polyphenols.

Regarding the nanoparticles, the thermogravimetric curve of PLA-HNTs + RO 1 presented a shift towards a closer value to that of PLA, indicating that RO trapped within the lumen of the nanoclays is not able to modify the polymeric matrix. By checking the curves for higher contents of HNTs loaded with RO, it can be seen that there is some shift to lower temperatures. The RO encapsulated within HNTs tend to remain the RO in the lumen, and thus avoids the thermal protection of PLA provided by RO. Meanwhile, HNTs induce a degradation temperature advance. The residual mass obtained for these samples corresponds to the amount of filler. Higher loading levels of HNTs (3 and 5 wt.%) lead to a small but significant (*p* < 0.05) reduction in the onset thermal degradation, but being thermally stable at temperatures considerably higher than the processing temperature used.

For microparticles, in the SEM and mechanical results section, it has been described that the apparent good dispersion and optimum matrix/oil/particle proportion of the PLA-DE + RO 1 can also be observed in the TGA curve. The PLA-DE + RO 1 onset degradation temperature turned out to be the highest, reaching ~350 °C. Small amounts of inorganic materials such as clays in a polymer matrix used to provide an increment in thermal stability [67]. Then in the PLA-DE + RO 3, there is a reduction in the $T_{10\%}$ (*p* < 0.05) and $T_{deg}$ values, probably due to the lesser interaction between PLA matrix and DE at this proportion, as already discussed in SEM results. As for the PLA-DE + RO 5, the modified polymer matrix by the excess of essential oil counteracts the positive effect of the DE particle interaction with the polymeric matrix, and the thermal stability resultant is close to that of the neat PLA.

#### 3.2.5. Antioxidant Activity

Considering that lipids are one of the main targets of oxidative reactions and, thus, the lipid oxidation process is responsible for a major problem in both natural and processed foodstuffs [10,14], the antioxidant activity of the developed RO-containing films was studied in direct contact with a fatty food simulant (simulant D1).

The antioxidant activity of *Rosmary officinalis* L. essential oil has been atributed to a cooperative results of its compositions [68]. Thus, the antioxidant activity was indirectly measured through the DPPH method, and the radical scavenging activity (RSA%) results are provided in Table 4. The RSA of pristine RO was 43.2 ± 1.9% showing values in the range of those observed in the literature [68,69].

All formulations containing RO, either directly added or encapsulated into HNTs or DE, showed antioxidant activity, highlighting that RO retains its antioxidant ability after thermal processing of the films by melt-extrusion followed by cast-film. The antioxidant activity of directly added RO to PLA films (PLA-RO) is comparable to other systems based on plasticized PLA incorporated with similar amounts of rosemary essential oil [70]. Therefore, the present results show that part of the RO was able to support the thermal processing and then also act as an effective antioxidant agent to protect packed food from oxidative processes. The antioxidant activity was higher in the films in which the RO was encapsulated, particularly in PLA-HNTs + RO 5 and PLA-DE + RO 1, since the EO directly added into the PLA matrix suffered thermal degradation due to the volatility of RO. While for nanoparticles, the higher loading levels of 5 wt.% lead to a higher antioxidant activity, as expected, in microparticles, the best results were observed for the lowest loading levels used of 1 wt.%. These findings are directly related to the already commented loss of RO from DE at higher loading levels, as it is a microparticle. Meanwhile, the RO in HNTs is better retained in the lumen of HNTs. Moreover, the results are in good agreement with the results of the onset degradation temperature tendency as the RO provided thermal protection due to the antioxidant activity.

It should be highlighted that the antioxidant activity of the encapsulated PLA-HNTs + RO 5 and PLA-DE + RO 1 is significantly higher (*p* < 0.05) than that of other systems based on PLA and rosemary essential oil as the fillers can protect the essential oil from thermal degradation.

### 3.3. Disintegration under Composting Conditions

**Figure 8:**
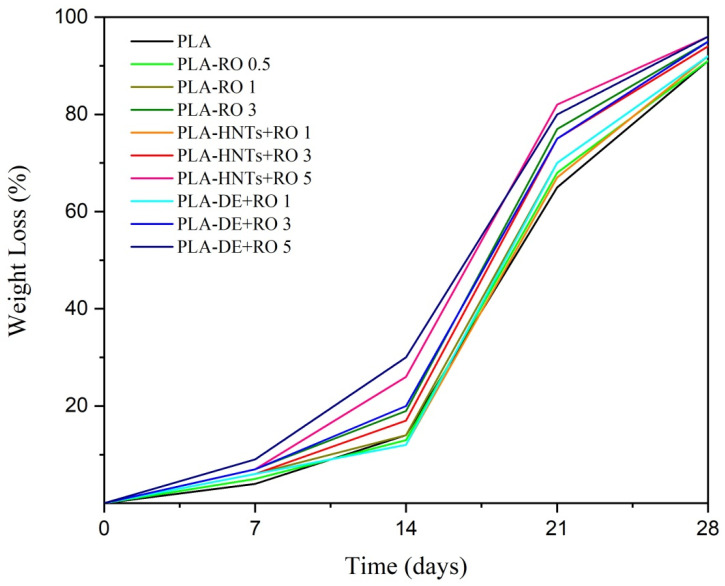
Degree of disintegration under composting conditions as a function of time of the developed films.

**Figure 9:**
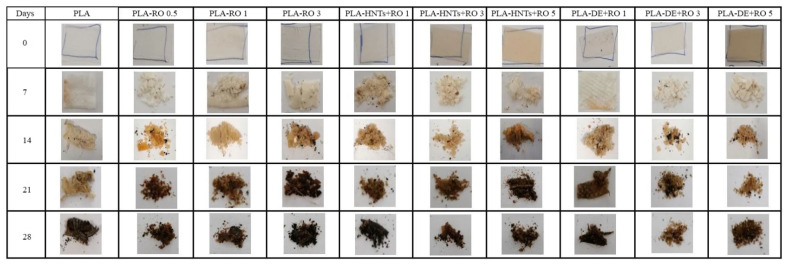
Visual appearance of films before and after different recovered days of disintegration under composting conditions.

The compostability of the films with RO and encapsulated RO in HNTs and DE was studied by a disintegration test in controlled composting conditions. The percentage weight loss of all the samples during the disintegration process is gathered in Figure 8, while the visual appearance of the recovered films at different disintegration days after exposure to the composting medium is shown in Figure 9. During the first week, normally related to the induction period, all the formulations lost the same mass of approximately 10%, which is slightly higher than neat PLA, showing that RO speeds up the disintegration phenomenon, as was already observed for other essential oils such as limonene [71] or carvacrol [12]. In the second week, significant differences started to be appreciated between the weight losses of the samples. In particular, formulations with the highest loading of both clays (PLA-HNTs + RO 5 and PLA-DE + RO 5) presented a disintegration rate close to 30%, whereas the rest did not exceed 20%. This increase in disintegration may be propinated by the increment of the moisture absorption resulting from the intrinsic hydrophilic character of the clays [72].

After 21 days, the disintegration rate of all the films was over 50%. It is worthy to note that the film with the highest content of non-encapsulated essential oil reached a weight loss similar to those just mentioned with the highest DE or HNTs content, thus evidencing that the essential oil also favors faster disintegradability due to the increased polymer chain mobility as a consequence of the already mentioned plasticizing effect of RO for the PLA matrix. Although more days were necessary in this case, it was due to the lower presence of RO in the PLA-RO 3 than in PLA-HUNTS + RO 5 and PLA-DE + RO 5. The increment of free volume, with more space between polymer chains that plasticization supposes, leads to an easier diffusion of water molecules and hence facilitates the hydrolysis activity to shorten the polymer chain and make the enzymatic disintegration promotion of the PLA matrix.

At the end of the burial period of 28 days, all the samples were almost disintegrated, with remaining mass of less than 10% (more than 90% of the weight was lost), which is the frequent value used in the literature to consider the materials disintegrated [10,73]. Figure 9 shows the variation of the appearance of the samples as the weeks progressed, where it can be seen how this small amount of mass remained, turning to a sand-kind aspect.

## 4. Conclusions

*Rosmarinus officinalis* (RO) essential oil was successfully encapsulated into DE microparticles as well as into HNTs nanoparticles and further used to produce PLA-based composites and nanocomposites, respectively. The materials were developed by melt extrusion followed by cast film, simulating the industrial processing conditions. Both DE and HNTs were able to protect the *Rosmarinus officinalis* (RO) from thermal degradation during processing, allowing to obtain films with antioxidant properties, as demonstrated by the antioxidant assays after the materials were exposed for 10 days to a fatty food simulant. The RO encapsulated into either DE or HNTs showed a reinforcing effect, particularly at 3 wt.% loading levels. However, from a structural point of view DE microparticles showed less adhesion to the PLA matrix than the smaller HNTs. In fact, DE at higher loading levels of 1 wt.% showed structural defects, while HNTs showed agglomerations at higher loading levels of 3 wt.%. Meanwhile, RO produced to a certain extent a plasticization effect, improving the interaction between DE or HNTs and the PLA polymeric matrix.

The films developed here showed their potential for sustainable films for biodegradable antioxidant food packaging applications.

## Figures and Tables

**Table 1:** Mechanical properties of the developed films obtained from tensile test measurements.

Film	*E* (MPa)	σ_y_ (MPa)	ε_b_ (%)
PLA	3700 ± 80 ^a,b,c^	38.5 ± 0.5 ^a^	2.3 ± 0.1 ^a,b^
PLA-RO 0.5	3880 ± 80 ^d,e^	39.8 ± 0.4 ^a^	1.7 ± 0.2 ^c^
PLA-RO 1	3980 ± 40 ^d^	38.4 ± 0.5 ^a^	1.2 ± 0.2 ^a^
PLA-RO 3	3120 ± 30 ^f^	26.0 ± 0.9 ^b^	3.6 ± 0.7 ^d^
PLA-HNTs + RO 1	3770 ± 40 ^a,e^	28.8 ± 0.2 ^b^	3.1 ± 0.3 ^b,d^
PLA-HNTs + RO 3	3590 ± 80 ^b^	36.6 ± 0.4 ^a^	2.7 ± 0.4 ^b^
PLA-HNTs + RO 5	2940 ± 60 ^g^	25.9 ± 0.2 ^b^	2.9 ± 0.3 ^b,d^
PLA-DE + RO 1	4110 ± 70 ^h^	46.9 ± 0.7 ^c^	4.8 ± 0.4 ^e^
PLA-DE + RO 3	4550 ± 60 ^i^	39.2 ± 0.4 ^a^	1.6 ± 0.2 ^c,d^
PLA-DE + RO 5	3670 ± 40 ^c^	29.7 ± 0.6 ^d^	4.3 ± 0.7 ^e^

^a–i^ Different letters within the same column show statistically significant differences between formulations (*p* < 0.05).

**Table 2:** DSC thermal parameters of the developed films.

Film	T_g_ (°C)	T_cc_ (°C)	ΔH_cc_ (J g^−1^)	T_m_ (°C)	ΔH_m_ (J g^−1^)	*χ*_c_ (%)
PLA	60.3 ± 0.8 ^a^	123.5 ± 1.3 ^a^	9.5 ± 0.8	153.5 ± 0.9 ^a^	20.7± 0.7	11.9 ± 0.1
PLA-RO 0.5	58.1 ± 1.4 ^a^	114.0 ± 0.9 ^b,c^	18.7 ± 0.7	153.6 ± 1.1 ^a^	27.3 ± 1.1	9.2 ± 0.2
PLA-RO 1	59.1 ± 1.2 ^a^	114.0 ± 1.1 ^b,c^	21.2 ± 0.9	147.8 ± 0.8 ^b,c^	29.4 ± 0.9	8.9 ± 0.2
PLA-RO 3	58.4 ± 1.4 ^a^	111.6 ± 1.4 ^b,c^	20.7 ± 0.6	148.2 ± 1.3 ^b,c^	26.0 ± 0.8	5.9 ± 0.7
PLA-HNTs + RO 1	58.1 ± 1.1 ^a^	111.3 ± 0.8 ^b,d^	16.6 ± 0.8	147.7 ± 1.1 ^c^	27.8 ± 0.7	12.1 ± 0.3
PLA-HNTs + RO 3	59.2 ± 1.1 ^a^	115.1 ± 1.2 ^b,c^	21.5 ± 0.8	148.7 ± 1.2 ^b,c^	22.8 ± 0.9	1.4 ± 0.4
PLA-HNT + RO 5	56.6 ± 1.3 ^a^	112.0 ± 1.1 ^b,e^	26.7 ± 0.9	147.7 ± 0.9 ^b,c^	27.7 ± 1.1	1.2 ± 0.3
PLA-DE + RO 1	58.9 ± 0.9 ^a^	118.1 ± 0.9 ^c^	11.0 ± 1.2	152.1 ± 0.8 ^a,b,d^	22.7 ± 0.8	12.6 ± 0.4
PLA-DE + RO 3	59.3 ± 1.2 ^a^	115.3 ± 1.3 ^b,c^	17.4± 1.1	148.2 ± 1.4 ^c,d^	22.9 ± 0.9	6.1 ± 0.2
PLA-DE + RO 5	57.7 ± 1.1 ^a^	108.6 ± 1.1 ^d,e^	12.8 ± 1.2	153.4 ± 1.2 ^a^	28.4 ± 0.7	17.6 ± 0.7

^a–e^ Different letters within the same column show statistically significant differences between formulations (*p* < 0.05).

**Table 3:** TGA results of the developed films.

Film	$T_{10\%}$ (°C)	$T_{deg}$	Residual Mass (%)
PLA	322 ± 1.2 ^a^	366.94 ± 1.1 ^a,b,c^	1.6 ± 0.2
PLA-RO 0.5	328. ± 1.2 ^b^	354.09 ± 0.9 ^a^	0.4 ± 0.3
PLA-RO 1	328.7 ± 0.9 ^b^	361.27 ± 1.1 ^a,b,c^	0.4 ± 0.2
PLA-RO 3	343.6 ± 1.1 ^c^	359.64 ± 0.7 ^a,b^	0.3 ± 0.2
PLA-HNTs + RO 1	322.1 ± 0.9 ^a^	353.29 ± 1.1 ^a^	0.6 ± 0.3
PLA-HNTs + RO 3	316.6 ± 1.5 ^d^	362.55 ± 1.4 ^a,b,c^	3.0 ± 0.1
PLA-HNTs + RO 5	313.8 ± 1.4 ^d^	372.61 ± 1.2 ^b,c^	5.0 ± 0.2
PLA-DE + RO 1	350.0 ± 1.1 ^e^	378.49 ± 1.2 ^c^	1.2 ± 0.3
PLA-DE + RO 3	306.2 ± 1.2 ^f^	360.81 ± 0.9 ^a,b,c^	2.3 ± 0.2
PLA-DE + RO 5	328.3 ± 0.9 ^b^	358.76 ± 1.1 ^a,b^	2.1 ± 0.1

^a–f^ Different letters within the same column show statistically significant differences between formulations (*p* < 0.05).

**Table 4:** RSA results of the developed films after 10 contact days in Simulant D1 at 40 °C.

Film	RSA (%)
PLA	-
PLA-RO 0.5	12.7 ± 2.5 ^a^
PLA-RO 1	22.1 ± 3.1 ^b^
PLA-RO 3	32.9 ± 1.9 ^c,d^
PLA-HNTs + RO 1	37.3 ± 2.1 ^e^
PLA-HNTs + RO 3	22.4 ± 3.2 ^b,f^
PLA-HNTs + RO 5	43.8 ± 1.6 ^e^
PLA-DE + RO 1	43.3 ± 1.4 ^e^
PLA-DE + RO 3	28.8 ± 1.3 ^d,f^
PLA-DE + RO 5	27.6 ± 0.9 ^g^

^a–g^ Different letters within the same column show statistically significant differences between formulations (*p* < 0.05).

## Data Availability

Data are contained within the article.
